# Concurrent wasting and stunting among marginalised children in Sana’a city, Yemen: a cross-sectional study

**DOI:** 10.1017/jns.2023.72

**Published:** 2023-08-04

**Authors:** Mansour Abdu Al-Taj, Abdulwahed Al Serouri, Anwar Mahmoud Al-Muradi, EzzAldeen Al-Dharhani, Nada Nabil Al-faeq, Fatima Mohammed Al-amodi, Muaadh Mohammed Abdulwahab, Ali Mujahed Nawfal, Manal Haza'a Alshemerry, Monia Abdullah Mujahed

**Affiliations:** 1 Department of Community Medicine, Faculty of Medicine and Health Sciences, Sana'a University, Sana'a, Yemen; 2 Field Epidemiology Training Programme, Ministry of Public Health and Population, Sana'a, Yemen; 3 Faculty of Medicine and Health Sciences, Sana'a University, Sana'a, Yemen

**Keywords:** Children, Concurrent wasting and stunting, Prevalence, Yemen

## Abstract

Concurrent wasting and stunting (WaSt) is a serious form of malnutrition among young children, particularly vulnerable groups affected by the conflict. Understanding the prevalence and risk factors of WaSt among vulnerable children is important to develop effective intervention measures to reduce the burden of WaSt. The present study aimed to identify the prevalence of and risk factors for WaSt among marginalised children aged 6–59 months in Sana’a city, Yemen. A community-based cross-sectional design was conducted on a total sample size of 450 marginalised children aged 6–59 months who lived at home with their mothers. Multivariable logistic regression analysis was performed and the prevalence of WaSt was found to be 10⋅7 %. Children aged 24–59 months were protected from WaSt (adjusted odds ratio (AOR) 0⋅40, 95 % confidence interval (CI) 0⋅21, 0⋅75). A higher prevalence of WaSt was associated with male sex (AOR 2⋅31, 95 % CI 1⋅13, 4⋅71), no history of being breastfed (AOR 3⋅57, 95 % CI 1⋅23, 10⋅39), acute diarrhoea (AOR 2⋅12, 95 % CI 1⋅12, 4⋅02) and family income sources of assistance from others (AOR 2⋅74, 95 % CI 1⋅08, 6⋅93) or salary work (AOR 2⋅22, 95 % CI 1⋅10, 4⋅47). Continued breast- and bottle-feeding were not associated with WaSt in children aged 6–23 months. Mothers’ age, education and work status, family size and drinking water source were not associated with WaSt. Overall, we found that the prevalence of WaSt among marginalised children remained high. Interventions to improve household income, hygienic conditions and child feeding practices are necessary to promote child growth.

## Introduction

Malnutrition remains a threat to children in developing countries and accounts for approximately 45 % of all child deaths^(1)^. Malnourished children are more prone to infections^(2–4)^. Wasting, stunting and underweight are the most common forms of malnutrition among children under age five. Wasting is commonly known when children have an abnormally low weight for their height. It is most common among children under 24 months^(5)^. It has an immediate effect on children and can cause death^(6)^. The condition known as stunting, on the other hand, occurs when children's height is abnormally low for their age. Compared to wasting, stunning has delayed or long-term developmental consequences^(7)^. When wasting and stunting combined, the condition is known as the concurrent wasting and stunting (WaSt)^(8–10)^.

WaSt is the most severe kind of malnutrition because of the combined effects of wasting and stunting^(11)^. The risk of death in children with WaSt was shown to be 12⋅75 times higher than the hazards associated with stunting and wasting separately, which were found to be 1⋅47 and 2⋅30 times higher, respectively^(12)^. Recent meta-analysis reported that the prevalence of WaSt ranges between 0 and 8 % in eighty-four countries, with the highest occurrence in the fragile and conflict-affected areas^(13)^. WaSt was substantially correlated with male sex^(8,9,14)^, age of 12–23 months, infection and maternal underweight^(8,15)^. A number of risk factors, including low birth weight^(16,17)^, poor socioeconomic status^(16,17)^ and maternal short stature^(17)^, have also been linked to both wasting and stunting. Though several studies highlighted the prevalence and risk factors of WaSt among children, the problem is not studied well in vulnerable groups in the war affected areas like Yemen.

Children's lives have been jeopardised by malnutrition in Yemen for many years^(18)^. Malnutrition prevalence has been significantly impacted by the ongoing conflict^(19,20)^. Food instability and limited access to health care, which are the main causes of malnutrition, have been exacerbated by the interruption of vital services, particularly food delivery and medical treatment^(20)^. Marginalised communities, who already faced considerable socioeconomic and health disparities, are the most affected^(21,22)^. Consequently, the malnutrition including WaSt is expected to be high among marginalised children. The size of WaSt among marginalised children in Yemen is not yet known. This dearth of information emphasises the necessity of a research to assess the prevalence of the issue and its risk factors. By providing such first estimates of the prevalence of WaSt among this cohort, this might considerably raise awareness of the problem and help in guiding policy decisions on health and nutrition measures.

## Methods

### Study design, setting and population

A cross-sectional study was conducted in Sana'a city between April and October 2021. The marginalised group here has been suffering from discrimination and poverty for decades^(21)^. These marginalised families live in several neighbourhoods in the city and its outskirts, scattered or in camps. Although no official statistics exist on the size of the population, the number is estimated to be between 0⋅5 and 3⋅5 million^(21)^. This marginalised community is considered one of the most vulnerable groups in Yemen^(22)^, and they usually have menial occupations. Households with at least one child aged 6–59 months living with their mother in camps were eligible to participate in this study. Children with chronic illness or malformation and families who refused to participate in the study were excluded.

### Sample size

A single-proportion formula was used to estimate the sample size at a 95 % confidence interval and a 5 % level of significance^(23)^. The prevalence of WaSt in similar studies ranged from 1 to 8 %. Since no previous WaSt studies from Yemen exist, the proportion of acute malnutrition from the national survey (16 %) was considered^(18)^. The initial sample size was 207 children; this number was then multiplied by two for the design effect to account for clustering and adjusted for an anticipated nonresponse rate of 10 %. The final sample size was 459 children aged 6–59 months.

### Sampling

The process of selecting the study samples was performed in two stages. In the first stage, four camps were randomly selected from the twelve identified in the study area. The sample size was proportionally distributed among the selected camps according to the estimated number of households of each camp. In the second stage, the required number of households inhabited by mothers and at least one child between 6 and 59 months of age was selected using the World Health Organization's guidelines for an immunisation survey expanded program^(24)^. If the household had more than one child aged 6–59 months, the youngest child was selected for assessment.

### Data collection

Data were collected from June 1 to 14, 2021, by 10 final-year medical students (five men and five women) who had been trained on how to ask questions and measure the length/height and weight of children. A structured questionnaire was used to collect data through direct interviews with mothers at their homes. The questionnaire consisted of three main sections: data related to the child (characteristics, feeding practices and anthropometric measurements), the mother (age, education and work status) and the household (family size, source of income, number of rooms, source of drinking water and presence of bathroom).

The ten data collectors were divided into five groups of two, who measured the length/height and weight of each child according to World Health Organization guidelines^(25)^. A stadiometer (Pimolchai board) was used to measure the children's length/height and a portable weighing scale (Seca, Model 874) was used to measure the children's weight. Weight was documented to the nearest 0⋅1 kg. Mothers were asked to remove their child's clothes and shoes and place them standing on the device installed on a flat floor. For young children who could not stand or refused to stand, the weight of the mother was measured first, then the weight of the mother with the child was measured and the child's weight was then subtracted from the total weight. For children aged 24–59 months, height was taken to the nearest 0⋅1 cm in a standing position, without shoes or head covers. Length was measured for children aged 6–23 months while they were in a supine position.

### Dependent and independent variables

The dependent variable was WaSt. According to the World Health Organization, a child is considered stunted, wasted or underweight if the Z-score of height-for-age (HAZ), weight-for-height (WHZ) and weight-for-age are ≤2 standard deviations (sd)^(26)^. A value between –2 and –3 for any of these variables indicates a moderate case, whereas values below –3 indicate a severe case. WaSt was considered to be present when the WHZ and HAZ were ≤2 sd. WaSt was measured as a binary variable and coded as follows: 1 = children with WaSt and 0 = children with no WaSt. ENA for SMART (2007) is used to compute WHZ, HAZ and WAZ values for each child.

The independent variables for the child-related demographic data included the child's age in months (classified into two groups: 6–23 or 24–59 months), sex (boy or girl) and birth order (first, second or third and higher). The child's age was taken based on the families’ available documents, such as immunisation cards or dates documented by a family member. If no documents existed, a calendar of major events and celebrations was used to help the mother recall the date she delivered the child.

All mothers were asked whether they had ever breastfed and whether they exclusively breastfed their child. ‘Ever breastfed’ was classified as a yes/no question, with children who had consumed their mother's milk designated ‘yes’ and children never fed with their mother's milk designated ‘no’. The child was considered exclusively breastfed if the mother stated that the child did not receive any sustenance during the first 6 months except breast milk and prescribed medicines^(27)^. Continued breast-feeding and bottle-feeding were assessed for children aged 6–23 months. Children 6–23 months of age who were fed from mothers’ milk on the day before the interview were marked ‘continued breast-feeding’. Children were deemed to be bottle-feeding if they consumed any food or drink, including their mother's milk, from a bottle with a nipple on the previous day^(27)^. Food diversity for each child was assessed by asking the mother to list all foods and beverages given to the child on the previous day. Food diversity was assessed using the World Health Organization recommended food groups, which include: breast milk, grains, roots, tubers, and plantains, pulses (beans, peas, and lentils), nuts, seeds, dairy products (e.g., milk, infant formula, yoghurt, and cheese), flesh foods (meat, poultry and organ meats), eggs, vitamin A-rich fruits and vegetables, and other fruits and vegetables. Children aged 6–23 months who consumed food and beverages from at least five of the eight recommended food groups on the previous day were considered ‘food diverse’^(27)^, while children aged 24–59 months were considered food diverse if they consumed at least four of the seven recommended food groups^(28)^. The child had acute diarrhoea if the mother reported that they had passed at least three loose or watery stools in 24 h at least once in the 2 weeks preceding the survey^(29)^.

Mothers’ characteristics were summarised by age in years and categorised into four groups (under 20, 20–29, 30–39 or 40 and older), educational level (no education, primary or secondary) and work status (employed or not). The household characteristics assessed included family income source, family size, number of rooms, source of drinking water and presence of a bathroom in the house. Family income source was divided into three categories: at least one family member earning daily wages, at least one family member earning a salary or the family receiving assistance from others. Family size was divided into families of five members or fewer and families of over five members. Drinking water source could include water tanks or others, such as buying treated water or collecting it from neighbours or wells.

### Statistical analysis

Proportions were used to describe categorical variables. To explore the factors related to WaSt, a χ^2^ test and Fisher's exact test were used. Multivariable logistic regression was performed to adjust for potential confounders, and AOR and 95 % CI were calculated. Mothers’ education was included *a priori*^(8)^. All variables associated with WaSt with a P-value of <0⋅10 in the bivariate analysis were checked for interactions and multicollinearity. The assessment of interaction was done using the likelihood ratio test, and multicollinearity was checked using the variance inflation factor. No evidence of interaction was found between the variables. The variance inflation factor was less than 1⋅1, indicating no multicollinearity. Stata 16 (Stata Corp., College Station, TX, USA) was used for data analysis.

### Ethics

This study was conducted according to the Declaration of Helsinki, and all procedures involving research study participants were approved by the ethical committee of Sana’a University. Verbal informed consent was obtained from all participants. Verbal consent was witnessed and formally recorded.

## Results

Nine families refused to participate in this study. Thus, 450 households were included in the analysis.

### Respondent characteristics

Table 1 shows the descriptive results for the children's characteristics, feeding practices and nutritional statuses. Nearly two-thirds of the children included were between 24 and 59 months old. The proportion of boys was higher than girls (58⋅0 % *v.* 42⋅0 %) and more than half of the children (60⋅0 %) were third or more in the family birth order. Around 5 % of children had never been fed with their mother's milk, and only 7⋅3 % were exclusively breastfed for 6 months. Among those aged 6–23 months, 60⋅6 % continued to breast-feed and 43⋅8 % were fed from a bottle with a nipple. Approximately 43 % of children consumed diverse food (as defined according to the described World Health Organization guidelines) in the 24 h before the interview. More than 40 % of children had had acute diarrhoea in the 2 weeks previous to the study.

**Table 1.** Child characteristic, feeding practices and nutritional status (*N* 450)

Variable name	Frequency	Percentage
Age of child (months)		
6–23	160	35⋅6
24–59	290	64⋅4
Sex of child		
Boy	261	58⋅0
Girl	189	42⋅0
Child’s birth order		
First	98	21⋅8
Second	82	18⋅2
Third or higher	270	60⋅0
Ever been breastfed		
Yes	427	94⋅9
No	23	5⋅1
Exclusively breastfed		
Yes	33	7⋅3
No	417	92⋅7
Continued breast-feeding (children 6–23 months)		
Yes	97	60⋅6
No	63	39⋅4
Bottle-feeding (children 6–23 months)		
Yes	70	43⋅8
No	90	56⋅2
Past 24-hour child food diversity		
Yes	194	43⋅1
No	256	56⋅9
Past 14-day diarrhoea		
Yes	190	42⋅2
No	260	57⋅8
Place of delivery		
Health facilities	117	26⋅0
Home	333	74⋅0
Nutritional status		
Moderate wasting (WHZ < –2 sd to –3 sd)	77	17⋅1
Severe wasting (WHZ < –3 sd)	43	9⋅6
Overall wasting (WHZ < –2 sd)	120	26⋅7
Moderate stunting (HAZ < –2 sd to –3 sd)	105	23⋅3
Severe stunting (HAZ < –3 sd)	128	28⋅4
Overall stunting (HAZ < –2 sd)	233	51⋅8
Moderate underweight (WAZ < –2 sd to –3 sd)	134	29⋅8
Severe underweight (WAZ < –3 sd)	90	20⋅0
Overall underweight (WAZ < –2 sd)	224	49⋅8
WaSt (WHZ < –2 sd and HAZ < –2 sd)	48	10⋅7

sd, standard deviation; WHZ, weight-for-height *Z*-score; HAZ, height-for-age *Z*-score; WAZ, weight-for-age *Z*-score; WaSt, concurrent wasting and stunting.

Table 1 shows the prevalence of wasting, stunting, underweight and WaSt. The prevalence of wasting, stunting and underweight status were 26⋅7, 51⋅8 and 49⋅8 %, respectively. Approximately 17 and 10 % of children had moderate and severe wasting, respectively. More than a quarter of children were severely stunted. The proportion of children who had WaSt was 10⋅7 %.

Table 2 shows the maternal and household characteristics. Approximately 45 % of mothers were 20–29 years old (44⋅2 %), more than half had no education (52⋅0 %), and the majority were housewives with no outside employment (91⋅3 %). More than half of the households had more than five members, and one-third of families lived in a single room. Almost 60 % of households were dependent on daily wages as their source of income, and 80⋅7 % relied on tanks as their source of drinking water.

**Table 2.** Maternal and household characteristics (*N* 450)

Variable name	Frequency	Percentage
Age of mother (years)		
<20	73	16⋅2
20–29	199	44⋅2
30–39	145	32⋅2
≥40	33	7⋅3
Education of mother		
None	234	52⋅0
Primary	175	38⋅9
Secondary	41	9⋅1
Mother’s work		
Yes	39	8⋅7
No	411	91⋅3
Family size		
≤5	198	44⋅0
>5	252	56⋅0
Number of rooms		
1	149	33⋅1
2	172	38⋅2
3	89	19⋅8
4 or more	40	8⋅9
Household source of income		
Daily wages	269	59⋅8
Salary	131	29⋅1
Assistance from others	50	11⋅1
Source of drinking water		
Tanks	363	80⋅7
Others	87	19⋅3
Bathroom in home		
Yes	340	75⋅6
No	110	24⋅4

### Distribution of child, maternal and household characteristics by WaSt

Table 3 shows the distribution of children's characteristics by WaSt. Children aged 6–23 months had a significantly higher prevalence of WaSt than those aged 24–59 months (16⋅2 % *v.* 7⋅6 %, P = 0⋅004). WaSt was significantly more common among boys than among girls (13⋅8 % *v.* 6⋅4 %, P = 0⋅012). Children who had never breastfed had a higher prevalence of WaSt than those who had (26⋅1 % *v.* 9⋅8 %, P = 0⋅014). WaSt was also more common among children with acute diarrhoea than among those without diarrhoea (13⋅7 % *v.* 8⋅5 %, P = 0⋅076). Birth order, exclusive breast-feeding, food diversity and place of delivery were not associated with WaSt. Among children aged 6–23 months, neither continued breast-feeding nor bottle-feeding was associated with WaSt.

**Table 3.** Distribution of child’s characteristics by WaSt (*N* 450)

Variable name	WaSt		*P*
	Yes *n* (%)	No *n* (%)	
Age of child (months)			0⋅004
6–23	26 (16⋅2)	134 (83⋅8)	
24–59	22 (7⋅6)	268 (92⋅4)	
Sex of child			0⋅012
Boy	36 (13⋅8)	225 (86⋅2)	
Girl	12 (6⋅4)	177 (93⋅6)	
Child’s birth order			0⋅883
First	10 (10⋅2)	88 (89⋅8)	
Second	10 (12⋅2)	72 (87⋅8)	
Third or higher	28 (10⋅4)	242 (89⋅6)	
Ever been breastfed			0⋅014
Yes	42 (9⋅8)	385 (90⋅2)	
No	6 (26⋅1)	17 (73⋅9)	
Exclusively breastfed			0⋅769^†^
Yes	4 (12⋅1)	29 (87⋅9)	
No	44 (10⋅6)	373 (89⋅4)	
Continued breast-feeding (children 6–23 months)			0⋅738
Yes	15 (15⋅5)	82(84⋅5)	
No	11 (17⋅5)	52 (82⋅5)	
Bottle-feeding (children 6–23 months)			0⋅305
Yes	9 (12⋅9)	61(87⋅1)	
No	17 (18⋅9)	73 (81⋅1)	
Past 24-hour child food diversity			0⋅102
Yes	26 (13⋅4)	168 (86⋅6)	
No	22 (8⋅6)	234 (91⋅4)	
Past 14-day diarrhoea			0⋅076
Yes	26 (13⋅7)	164 (86⋅3)	
No	22 (8⋅5)	238 (91⋅5)	
Place of delivery			0⋅606
Health facilities	11 (9⋅4)	106 (90⋅6)	
Home	37 (11⋅1)	296 (88⋅9)	

WaSt, concurrent wasting and stunting; *n*, number; %, percent; P, P-value; ^†^, Fisher's exact test was used.

Table 4 shows that WaSt was significantly more common among children whose mothers worked outside the home than those whose mothers were housewives (20⋅5 % *v.* 9⋅7 %, P = 0⋅037). The prevalence of WaSt was over twice as high among children whose families depended on assistance (16⋅0 %) or salary work (15⋅3 %) compared to families relying on daily wages (7⋅4 %, P = 0⋅025).

**Table 4.** Distribution of maternal and household characteristics by WaSt (*N* 450)

Variable name	WaSt		*P*
	Yes *n* (%)	No (%)	
Age of mother (years)			0⋅483^†^
<20	11 (15⋅1)	62 (84⋅9)	
20–29	22 (11⋅1)	177 (88⋅9)	
30–39	12 (8⋅3)	133 (91⋅7)	
≥40	3 (9⋅1)	30 (90⋅9)	
Education of mother			0⋅947^†^
None	24 (10⋅3)	210 (89⋅7)	
Primary	20 (11⋅4)	155 (88⋅6)	
Secondary	4 (9⋅8)	37 (90⋅2)	
Mother’s work			0⋅037
Yes	8 (20⋅5)	31 (79⋅5)	
No	40 (9⋅7)	371 (90⋅3)	
Family size			0⋅133
≤5	26 (13⋅1)	172 (86⋅9)	
>5	22 (8⋅7)	230 (91⋅3)	
Number of rooms			0⋅306^†^
1	18 (12⋅1)	131 (87⋅9)	
2	15 (8⋅7)	157 (91⋅3)	
3	13 (14⋅6)	76 (85⋅4)	
4 or more	2 (5⋅0)	38 (95⋅0)	
Household source of income			0⋅025
Daily wages	20 (7⋅4)	249 (92⋅6)	
Salary	20 (15⋅3)	111 (84⋅7)	
Assistance from others	8 (16⋅0)	42 (84⋅0)	
Source of drinking water			0⋅378
Tanks	41 (11⋅3)	322 (88⋅7)	
Others	7 (8⋅0)	80 (92⋅0)	
Bathroom in home			0⋅794
No	11 (10⋅0)	99 (90⋅0)	
Yes	37 (10⋅9)	303 (89⋅1)	

WaSt, concurrent wasting and stunting; *n*, number; %, percent; P, P-value; ^†^ Fisher's exact test was used.

### Factors associated with WaSt

In the multivariable analysis, children aged between 24 and 59 months remained protected against WaSt (AOR 0⋅40, 95 % CI 0⋅21, 0⋅75). The odds of WaSt were higher among boys (AOR 2⋅31, 95 % CI 1⋅13, 4⋅71) than among girls and among those who had never been breastfed (AOR 3⋅57, 95 % CI 1⋅23, 10⋅39) compared to those who had been. Children who had had diarrhoea in the previous two weeks to the study had higher occurrence of WaSt than those with no diarrhoea (AOR 2⋅12, 95 % CI 1⋅12, 4⋅02). Compared to children whose family source of income was daily wages, children whose family source of income was assistance from others (AOR 2⋅74, 95 % CI 1⋅08, 6⋅93) or salary work (AOR 2⋅22, 95 % CI 1⋅10, 4⋅47) had higher occurrence of WaSt. The positive association between the mother’s work and child WaSt in the bivariate analysis became borderline after adjusting for the potential confounders, and the 95 % CI included 1 (AOR 2⋅45, 95 % CI 0⋅97, 6⋅20). Maternal education was not associated with WaSt in children (Table 5).

**Table 5.** Multivariable analysis for the risk factors of WaSt among marginalised children in Sana’a, Yemen

Variable name	Crude odds ratio (95 % CI)	Adjusted odds ratio (95 % CI)
Age of child (months)		
6–23	Ref.	
24–59	0⋅42 (0⋅23–0⋅77)a	0⋅40 (0⋅21–0⋅75)a
Sex of child		
Girl	Ref.	
Boy	2⋅36 (1⋅19–4⋅67)a	2⋅31 (1⋅13–4⋅71)a
Ever been breastfed		
Yes	Ref.	
No	3⋅24 (1⋅21–8⋅65)a	3⋅57 (1⋅23–10⋅39)a
Past 14-day diarrhoea		
No	Ref.	
Yes	1⋅72 (0⋅94–3⋅13)	2⋅12 (1⋅12–4⋅02)a
Mother’s work		
No	Ref.	
Yes	2⋅39 (1⋅03–5⋅56)a	2⋅45 (0⋅97–6⋅20)
Education of mother		
None	Ref.	
Primary	1⋅13 (0⋅60–2⋅12)	1⋅08 (0⋅55–2⋅13)
Secondary	0⋅95 (0⋅31–2⋅88)	1⋅03 (0⋅31–3⋅38)
Household source of income		
Daily wages	Ref.	
Salary	2⋅24 (1⋅16–4⋅34)a	2⋅22 (1⋅10–4⋅47)a
Assistance from others	2⋅37 (0⋅98–5⋅73)	2⋅74 (1⋅08–6⋅93)a

CI, confidence interval; Ref., reference value; a, statistically significant association at P < 0⋅05.

## Discussion

The present study revealed that WaSt is prevalent among marginalised children in Sana’a city, Yemen. We found that the strongest predicators for WaSt among these children were being male, being from a household dependent on salary or income assistance from others and having acute diarrhoea. The WaSt prevalence among studied marginalised Yemeni children (10⋅7 %) was higher than elsewhere (i.e., as compared to 1⋅4 % in Ghana^(16)^, 4⋅9 % in Uganda^(8)^, 5⋅8 % in Ethiopia^(14)^ and 6⋅2 % in Senegal^(9)^). Several possible explanations exist for the high rate found in this study compared to others. First, our study population was more vulnerable than those in Ghana, Uganda, Ethiopia and Senegal. Second, this study was conducted during wartime and the worst humanitarian crisis in the world. As a result of the conflict, salaries had been cut off for government employees, including these marginalised people since 2016, with employees only receiving half a salary every 4–6 months. This was accompanied by a sharp increase in food and medicine prices. Finally, the lack of access to safe drinking water and sanitation, as well as the high illiteracy rate of parents in this community, may have increased the prevalence of WaSt.

Consistent with studies conducted in Ethiopia^(14)^ and Senegal^(9)^, this study found that children aged 6–23 months were at a higher risk of WaSt than older children aged between 24 and 59 months. In Uganda, children aged 36–59 months were protected from WaSt compared to those aged 6–11 months^(8)^. A recent meta-analysis reported a decline in the proportion of WaSt after 24 months^(13)^. Thus, 0–24 months is a critical period during which improper infant feeding practices^(30–33)^, as well as children's contact with the environment^(34)^, increase their chances of catching infections, leading to malnutrition. Reports indicate the deterioration of child nutritional status, on average, from 0 to 24 months^(35,36)^.

In line with similar studies from Uganda^(8)^, Senegal^(9)^ and Ethiopia^(14)^, our findings revealed that boys were at higher risk of WaSt than girls. Similarly, a study by Niger reported that boys aged <24 months were more likely to have WaSt^(17)^. The mechanism of the effects of sex on malnutrition remains unknown.

Regarding child feeding practices, no history of breast-feeding was a strong predictor of WaSt. Breast milk contains many vitamins, minerals and nutrients^(37–39)^ necessary for child growth. It also reduces the risk of infection in children and supports their immune systems^(37–39)^. Surprisingly, exclusive breast-feeding during the first 6 months was not associated with WaSt, possibly due to the low proportion of exclusive breast-feeding practices in the marginalised community. Also unexpectedly, the study did not find any association between children’s food diversity and WaSt. Thus, the findings of our study are inconsistent with a systematic review that found that not eating a varied diet increases the risk of all nutritional deficits, including wasting, stunting and underweight status^(40)^. The dietary diversity score was developed as a population-level proxy indicator for micronutrient malnutrition. Our findings reflect that a child's food intake in the 24 h prior to interview may not be sufficient to define a relationship between dietary diversity and WaSt.

Here, acute diarrhoea was linked to WaSt, which is consistent with previous findings^(8,15)^. A previous report from Bangladesh indicated that dehydrating diarrhoea was associated with severe wasting in severely stunted children^(41)^. Diarrhoea is both a cause and an effect of malnutrition. A recent systematic review reported that wasting is among the leading risk factors for diarrhoea, and that prevention of child wasting could prevent death from diarrhoea^(42)^. Children with diarrhoea become thinner due to loss of body weight, malabsorption and loss of protein and other nutrients^(43)^. In addition, diarrhoea has been reported to cause stunted growth in children aged under 24 months^(44)^.

Mothers’ education was not a risk factor for WaSt among children, unlike in the Ugandan's study, which found that low maternal education was associated with WaSt^(8)^. However, this finding is in line with that of an Ethiopian study^(14)^. The restricted prospects for paid labour and households that experience food insecurity may limit educated women's capacity to offer nutritious food for their children and to contribute to the development of WaSt, despite the fact that they may be more knowledgeable about child feeding practices and dietary diversity. Moreover, illiteracy is common among marginalised women, and some may only complete the most basic education (e.g., primary school). Therefore, the higher prevalence of illiteracy among these marginalised people may explain this lack of relationship. Maternal work was associated with child WaSt in the bivariate analysis; however, in the multivariable analysis, this relationship became insignificant, possibly due to the influence of income source on WaSt.

The present study's findings revealed that children from families whose source of income was salary work or assistance from others were at higher risk of WaSt than their daily-wage counterparts. A possible explanation is that, while working for daily wages may be unstable, the financial return, which is approximately $10 per day, is much higher than the income of a family that depends on a monthly salary (with a salary of approximately $40 per month) or assistance from others. Therefore, those who work for daily wages can better provide food and health care for their children than those who depend on other sources. Poverty contributes to household food insecurity, which causes an increase in malnutrition among children^(45)^. Previous studies have found that children from middle class, poor and the poorest households are at higher risk of WaSt than those from rich families^(8,15)^.

The majority of these marginalised populations lack access to basic services and living conditions^(22)^. However, our bivariate analysis showed no association between child WaSt and family size, number of rooms owned by families, presence of a bathroom or source of drinking water.

The present study's main limitation was that not all marginalised families in Sana'a city live in the camps where the study was conducted. Thus, our findings may not be generalisable to the city's entire marginalised population. Reaching all such families in the city would have required a large-scale, citywide survey, beyond the scope of this study. An additional limitation of the study was that it was carried out only among marginalised people where the number of mothers with paid work and those with any levels of education was small, and many of the households in our study had poor living conditions, which may have limited our ability to detect significant associations between these variables and WaSt. Further comparative studies with other community groups are necessary to determine the risk factors for WaSt. This study was also subject to recall bias.

## Conclusion

The prevalence of WaSt among marginalised children in Sana'a, Yemen, was high. The strongest predicators of WaSt in marginalised children were being aged 6–23 months, being male, having never been breastfed, having acute diarrhoea and household source of income being salary work or assistance from others. Urgent livelihood and hygiene interventions are recommended to mitigate the WaSt of vulnerable children in Yemen.

## References

[1] World health organization (2021) Malnutrition. https://www.who.int/news-room/fact-sheets/detail/malnutrition (accessed May 2022).

[2] Ibrahim MK, Zambruni M, Melby CL, et al. (2017) Impact of childhood malnutrition on host defense and infection. Clin Microbiol Rev 30, 919–971. 28768707 10.1128/CMR.00119-16 PMC5608884

[3] Walson JL & Berkley JA (2018) The impact of malnutrition on childhood infections. Curr Opin Infect Dis 31, 231–236. 29570495 10.1097/QCO.0000000000000448 PMC6037284

[4] Rytter MJH, Kolte L, Briend A, et al. (2014) The immune system in children with malnutrition – a systematic review. PLoS ONE 9, e105017. 25153531 10.1371/journal.pone.0105017 PMC4143239

[5] Karlsson O, Kim R, Guerrero S, et al. (2022) Child wasting before and after age two years: a cross-sectional study of 94 countries. EClinicalMedicine 46, 101353–101353. 35360149 10.1016/j.eclinm.2022.101353 PMC8961190

[6] Schwinger C, Golden MH, Grellety E, et al. (2019) Severe acute malnutrition and mortality in children in the community: comparison of indicators in a multi-country pooled analysis. PLoS ONE 14, e0219745. 31386678 10.1371/journal.pone.0219745 PMC6684062

[7] Alam MA, Richard SA, Fahim SM, et al. (2020) Impact of early-onset persistent stunting on cognitive development at 5 years of age: results from a multi-country cohort study. PLoS ONE 15, e0227839. 31978156 10.1371/journal.pone.0227839 PMC6980491

[8] Obeng-Amoako GA O, Karamagi CAS, Nangendo J, et al. (2021) Factors associated with concurrent wasting and stunting among children 6–59 months in Karamoja, Uganda. Matern Child Nutr 17, e13074. 32830434 10.1111/mcn.13074 PMC7729532

[9] Garenne M, Myatt M, Khara T, et al. (2019) Concurrent wasting and stunting among under-five children in Niakhar, Senegal. Matern Child Nutr 15, e12736. 30367556 10.1111/mcn.12736 PMC6587969

[10] Briend A, Khara T & Dolan C (2015) Wasting and stunting–similarities and differences: policy and programmatic implications. Food Nutr Bull 36, S15–S23. 25902610 10.1177/15648265150361S103

[11] Thurstans S, Sessions N, Dolan C, et al. (2022) The relationship between wasting and stunting in young children: a systematic review. Matern Child Nutr 18, e13246. 34486229 10.1111/mcn.13246 PMC8710094

[12] Myatt M, Khara T, Schoenbuchner S, et al. (2018) Children who are both wasted and stunted are also underweight and have a high risk of death: a descriptive epidemiology of multiple anthropometric deficits using data from 51 countries. Arch Public Health 76, 28–28. 30026945 10.1186/s13690-018-0277-1 PMC6047117

[13] Khara T, Mwangome M, Ngari M, et al. (2018) Children concurrently wasted and stunted: a meta-analysis of prevalence data of children 6-59 months from 84 countries. Matern Child Nutr 14, e12516. 28944990 10.1111/mcn.12516 PMC5901398

[14] Roba AA, Assefa N, Dessie Y, et al. (2021) Prevalence and determinants of concurrent wasting and stunting and other indicators of malnutrition among children 6-59 months old in Kersa, Ethiopia. Matern Child Nutr 17, e13172. 33728748 10.1111/mcn.13172 PMC8189198

[15] Sahiledengle B, Agho KE, Petrucka P, et al. (2023) Concurrent wasting and stunting among under-five children in the context of Ethiopia: a generalised mixed-effects modelling. Matern Child Nutr 19, e13483. 36757269 10.1111/mcn.13483 PMC10019057

[16] Saaka M & Galaa SZ (2016) Relationships between wasting and stunting and their concurrent occurrence in Ghanaian preschool children. J Nutr Metab 2016, 4654920–4654920. 27379184 10.1155/2016/4654920 PMC4917721

[17] Kohlmann K, Sudfeld CR, Garba S, et al. (2021) Exploring the relationships between wasting and stunting among a cohort of children under two years of age in Niger. BMC Public Health 21, 1713–1713. 34548050 10.1186/s12889-021-11689-6 PMC8454021

[18] Ministry of Public Health and Population, Central Statistical Organization, The Pan Arab Program for Family Health, ICF International (2015) Yemen National Health and Demographic Survey 2013. https://dhsprogram.com/pubs/pdf/FR296/FR296.pdf (accessed February 2021).

[19] El Bcheraoui C, Jumaan AO, Collison ML, et al. (2018) Health in Yemen: losing ground in war time. Global Health 14, 42. 29695301 10.1186/s12992-018-0354-9 PMC5918919

[20] World health organization (2021) Acute malnutrition threatens half of children under five in Yemen in 2021: UN. https://www.who.int/news/item/12-02-2021-acute-malnutrition-threatens-half-of-children-under-five-in-yemen-in-2021-un (accessed October 2021).

[21] United Nations High Commissioner for Refugees (2021) Yemen's ‘marginalized ones’ endure hunger, displacement. https://www.unhcr.org/news/stories/2021/2/601bcbad4/yemens-marginalized-ones-endure-hunger-displacement.html (accessed April 2023).

[22] United Nations Children's Fund (2014) Yemen: Breaking social barriers to reach marginalized communities. https://www.unicef.org/mena/press-releases/yemen-breaking-social-barriers (accessed October 2022).

[23] Daniel WW & Cross CL (2018) Biostatistics: A Foundation for Analysis in the Health Sciences. Hoboken: Wiley.

[24] World Health Organization (2020) Training for Mid-Level Managers (MLM): Module 7: The EPI Coverage Survey. Geneva: World Health Organization.

[25] World Health Organization (2008) Training Course on Child Growth Assessment. https://apps.who.int/iris/bitstream/handle/10665/43601/9789241595070_B_eng.pdf (accessed October 2022).

[26] World Health Organization (2019) Nutrition Landscape Information System (NLIS) country profile indicators: interpretation guide. https://apps.who.int/iris/bitstream/handle/10665/332223/9789241516952-eng.pdf.

[27] World Health Organization & United Nations Children's Fund (2021) Indicators for assessing infant and young child feeding practices: definitions and measurement methods. https://creativecommons.org/licenses/by-nc-sa/3.0/igo.

[28] World Health Organization (2010) Indicators for assessing infant and young child feeding practices part 3: country profiles. https://apps.who.int/iris/bitstream/handle/10665/44368/9789241599757_eng.pdf.

[29] United Nations Children's Fund & World Health Organization (2017) Diarrhoea. Why children are still dying and what can be done. 2009. Retrieved from: http://apps.who.int/iris/bitstream/10665/44174/1/978924 1598415_eng. pdf.

[30] Ahmed KY, Page A, Arora A, et al. (2020) Associations between infant and young child feeding practices and acute respiratory infection and diarrhoea in Ethiopia: a propensity score matching approach. PLoS ONE 15, e0230978. 32236145 10.1371/journal.pone.0230978 PMC7112197

[31] Ogbo FA, Nguyen H, Naz S, et al. (2018) The association between infant and young child feeding practices and diarrhoea in Tanzanian children. Trop Med Health 46, 2. 29422772 10.1186/s41182-018-0084-y PMC5791185

[32] Ogbo FA, Agho K, Ogeleka P, et al. (2017) Infant feeding practices and diarrhoea in Sub-Saharan African countries with high diarrhoea mortality. PLoS ONE 12, e0171792. 28192518 10.1371/journal.pone.0171792 PMC5305225

[33] Hajeebhoy N, Nguyen PH, Mannava P, et al. (2014) Suboptimal breastfeeding practices are associated with infant illness in Vietnam. Int Breastfeeding J 9, 12. 10.1186/1746-4358-9-12 PMC4121620 25097662

[34] Prentice AM (2020) Environmental and physiological barriers to child growth and development. Nestle Nutr Inst Workshop Ser 93, 125–132. 31991426 10.1159/000503349

[35] Prentice AM, Ward KA, Goldberg GR, et al. (2013) Critical windows for nutritional interventions against stunting. Am J Clin Nutr 97, 911–918. 23553163 10.3945/ajcn.112.052332 PMC3628381

[36] Stein AD, Wang M, Martorell R, et al. (2010) Growth patterns in early childhood and final attained stature: data from five birth cohorts from low- and middle-income countries. Am J Hum Biol 22, 353–359. 19856426 10.1002/ajhb.20998 PMC3494846

[37] Andreas NJ, Kampmann B & Mehring Le-Doare K (2015) Human breast milk: a review on its composition and bioactivity. Early Hum Dev 91, 629–635. 26375355 10.1016/j.earlhumdev.2015.08.013

[38] Mosca F & Giannì ML (2017) Human milk: composition and health benefits. Pediatr Med Chir 39, 155. 28673076 10.4081/pmc.2017.155

[39] Lokossou GAG, Kouakanou L, Schumacher A, et al. (2022) Human breast milk: from food to active immune response with disease protection in infants and mothers. Front Immunol 13, 849012–849012. 35450064 10.3389/fimmu.2022.849012 PMC9016618

[40] Molani Gol R, Kheirouri S & Alizadeh M (2022) Association of dietary diversity with growth outcomes in infants and children aged under 5 years: a systematic review. Journal of Nutrition Education and Behavior 54, 65–83. 35000681 10.1016/j.jneb.2021.08.016

[41] Nuzhat S, Shahunja KM, Shahid ASMSB, et al. (2020) Diarrhoeal children with concurrent severe wasting and stunting compared to severe wasting or severe stunting. Trop Med Int Health 25, 928–935. 32446268 10.1111/tmi.13446

[42] Troeger C, Blacker BF, Khalil IA, et al. (2018) Estimates of the global, regional, and national morbidity, mortality, and aetiologies of diarrhoea in 195 countries: a systematic analysis for the Global Burden of Disease Study 2016. Lancet Infect Dis 18, 1211–1228. 30243583 10.1016/S1473-3099(18)30362-1 PMC6202444

[43] Iddrisu I, Monteagudo-Mera A, Poveda C, et al. (2021) Malnutrition and gut microbiota in children. Nutrients 13, 2727. 34444887 10.3390/nu13082727 PMC8401185

[44] Guerrant RL, DeBoer MD, Moore SR, et al. (2013) The impoverished gut – a triple burden of diarrhoea, stunting and chronic disease. Nat Rev Gastroenterol Hepatol 10, 220–229. 23229327 10.1038/nrgastro.2012.239 PMC3617052

[45] Food and Agriculture Organization, International Fund for Agricultural Development, United Nations Children's Fund, et al. (2020) The State of Food Security and Nutrition in the World 2020. Transforming Food Systems for Affordable Healthy Diets. Rome: FAO.

